# Multifractal-based nuclei segmentation in fish images

**DOI:** 10.1007/s10544-017-0208-x

**Published:** 2017-08-03

**Authors:** Nikola Reljin, Marijeta Slavkovic-Ilic, Coya Tapia, Nikola Cihoric, Srdjan Stankovic

**Affiliations:** 1 Academic Technology Services, Princeton University, Princeton, NJ USA; 2 Innovation Center of the School of Electrical Engineering, University of Belgrade, Belgrade, Serbia; 3 Division of Clinical Pathology, Institute of Pathology, University of Bern, Bern, Switzerland; 4 Department of Radiation Oncology, Bern University Hospital, University of Bern, Bern, Switzerland; 5 School of Electrical Engineering, University of Belgrade, Belgrade, Serbia

**Keywords:** Breast cancer, Fluorescence *in-situ* hybridization, HER2, Image processing, Nuclei segmentation, Fractal and multifractal analyses, Holder exponents

## Abstract

The method for nuclei segmentation in fluorescence *in-situ* hybridization (FISH) images, based on the inverse multifractal analysis (IMFA) is proposed. From the blue channel of the FISH image in RGB format, the matrix of Holder exponents, with one-by-one correspondence with the image pixels, is determined first. The following semi-automatic procedure is proposed: initial nuclei segmentation is performed automatically from the matrix of Holder exponents by applying predefined hard thresholding; then the user evaluates the result and is able to refine the segmentation by changing the threshold, if necessary. After successful nuclei segmentation, the HER2 (human epidermal growth factor receptor 2) scoring can be determined in usual way: by counting red and green dots within segmented nuclei, and finding their ratio. The IMFA segmentation method is tested over 100 clinical cases, evaluated by skilled pathologist. Testing results show that the new method has advantages compared to already reported methods.

## Introduction

In developed countries the second cause of mortality is that of the cancer, just after the cardiovascular diseases as noted in World Health Organization (WHO) reports ( 2014). For women, the most common cause of death is the breast cancer, which makes about 23% of all cancers, with high mortality rate of around 14%. Very often (in 20% to 25% cases), this cancer is followed by the over-expression of the glycoprotein HER2 (human epidermal growth factor receptor 2, also called HER2/*neu*, or c-erb-B2) (Akiyama et al. 1986) which is located on the surface of breast cells and is responsible for the cell growth, differentiation and division. The HER2 receptor is controlled by HER2 gene located at the cell’s nucleus, at chromosome 17 near to its centromere (CEP-17). In normal cases cell has two copies of HER2 gene, growth signals are relatively weak and controllable, and cell’s membrane contains 20,000 to 50,000 HER2 molecules (Arnold et al. 2008). In some cases HER2 gene is amplified, having more than two copies. This leads to increased synthesis of HER2 protein - protein level may be even 100 times or more of those in normal cells, as noted in Venter et al. (1987). This state, known as HER2 protein over-expression, can drive the uncontrolled cell’s division, producing thus an aggressive tumor growth. Such breast cancers, referred as HER2 positive, are followed by high metastatic activity and a poor clinical prognosis: higher rate of recurrence and mortality (Slamon et al. 1987; Andrulis et al. 1998).

Fortunately, HER2 positive tumors are promising target for the therapy with the humanized monoclonal antibody known as *Herceptin* (chemical name *trastuzumab*, Genentech, San Francisco, CA). The Herceptin induces rapid removal of HER2 from the cell surface, thereby dramatically reducing the risk of recurrence and mortality, even in advanced cancer cases (Ross et al. 2009). This drug is effective against HER2-positive invasive cancers, but in addition to therapy being expensive, it can be useless if is applied for HER2 negative cases. Moreover, wrong therapy may even produce serious side effects and survival problems. Therefore, it is of great importance to accurately determine the HER2 positivity before applying Herceptin therapy (Slamon et al. 2011).

According to the guidelines and recommendations stated from the American Society of Clinical Oncology (ASCO) and the College of American Pathologists (CAP) (2013), the two FDA-approved methods for testing HER2 positivity are used in clinical praxis: the *Immunohistochemistry* (IHC) and *In-situ hybridization* (ISH). Both methods analyze the histopathology samples of the breast tissue, stained on an appropriate way. The IHC is of the qualitative nature, estimating the amount of HER2 protein on the cancer cell surface. The ISH method permits quantitative scoring of the HER2 status by measuring the number of HER2 genes copies on the chromosome within the cell’s nucleus.

The IHC method is routinely used in laboratories due to its simplicity, relatively low cost and the use of standard light microscope. The method is based on the staining reaction between HER2 proteins and an antibody on slides of breast tissue. After reaction, from the amount of perceptible membrane staining it is relatively easy to classify observed samples as HER2 negative or HER2 positive. Cases characterized by no or barely membrane staining observed in less than 10% of tumor cells are scored 0 or 1+ and are HER2 negative. Conversely, when strong complete membrane staining is observed in more than 30% of tumor cells, this case is scored 3+ and assumed HER2 positive. Unfortunately, many of observed samples are on the borderline, scored as 2+ (meaning as weakly positive), and need additional evaluation. The main difficulty in scoring borderline cases is that the IHC method is subjective, and different pathologists may use slightly different criteria to decide whether the results are positive or negative, although some automated procedures for HER2 scoring from IHC are reported, for instance in Hall et al. (2008).

With the ISH method, selective staining of particular DNA sequences is obtained allowing the detection, analysis, and quantification of specific abnormalities within interphase nuclei. Historically, the first of ISH methods use fluorescent markers: specific fluorescent probes that bind to particular parts of the chromosome (Arnold et al. 2008). Such method, known as the FISH (fluorescent ISH), enables the precise scoring of HER2 status without the need of cell culturing (which is necessary step in IHC), thus it can be applied to the analysis of any cytological or histological samples. Typical FISH employ two fluorescent dyes: Spectrum Orange (or Texas Red) for staining HER2 genes, and FITC (fluorescein-5-isothiocyanate) for staining CEP-17 centromere. After exciting the stained sample by light source, the fluorescent probe emits particular color. Under the fluorescence microscope HER2 genes will be visible as red and CEP-17 centromeres as green dots.

Standard procedure of evaluating HER2 status from FISH images is based on manual counting the red and green dots inside well defined and non-overlapping interphase nuclei (20 nuclei per tissue specimen is recommended) and calculating accurately the HER2 status from the average ratio of red-green dots (meaning, scoring the ratio HER2/CEP-17). For better recognizing cell nuclei (where red/green dots should be counted), the slides are treated also with the third fluorochrome known as the DAPI (4′,6-diamidino-2-phenylindole). The DAPI is bound to the cell nuclei and emits blue color after activating by light source. Since the fluorescence effect fades relatively quickly, the fluorescence microscope usually is equipped with digital camera, for recording obtained image enabling further (off-line) examination (Arnold et al. 2008).

Precise and detailed recommendations and guidelines for HER2 testing and scoring are given by ASCO/CAP (2013) and embedded in commercially available probe kits, for instance in HER2 FISH pharmDx™ Assay Kit, Dako (2010) and PathVysion HER-2 DNA Probe Kit (Abbott Molecular) (2013). In short, we can stress out that if HER2/CEP-17 ratio is greater than 2.2 the case is assumed as HER2 positive, while the case is HER2 negative if this ratio is less than 1.8. Cases between these values are suspicious and need special attention and additional examinations (Skaland et al. 2008).

Relatively recently, another two ISH-based methods are also accepted from the FDA as diagnostic tools for determining HER2 status (Jacquemier et al. 2013). These methods are the chromogenic ISH (CISH), which is the dual-probe method (using two colors: red for HER2 genes and blue for CEP-17 centromeres), and silver ISH (SISH), which is single-probe method (only HER2 genes are colored as black dots). Methods for staining and hybridization are simpler than in case of FISH. Also, specimens are stable providing permanent glass slide and the use of standard light microscope for visualization of HER2 gene copies. From these reasons CISH and SISH techniques are very promising for HER2 positivity testing. Note also that very recently the new micrometer-scale interphase FISH (so called μFISH) is described in Huber et al. (2016). This method is compatible with the standard FISH, but the procedure is significantly faster and enables spatially multiplexed FISH. However, even today, the dual-color FISH technique is still assumed as a “golden standard” for HER2 status scoring (Dybdal et al. 2005; Tapia et al. 2007; Perez et al. 2014).

The paper is organized as follows. In Section 2 the problem statement and brief review of known techniques for cell nuclei segmentation are presented. Section 3 introduces the concept of fractal and multifractal (MF) analyses as a powerful ways for describing, analyzing and evaluating complex structures, phenomena and signals in general, and their application in image processing as well. One of the main benefits of using MF analysis is that it permits to describe observed structure in local and global sense mutually. Section 4 considers the possibility of the use of MF analysis (MFA) for cell nuclei segmentation, and the new method based on the so called IMFA (inverse multifractal analysis) algorithm is proposed. It is shown that the IMFA method is less sensitive to inhomogeneous slide illumination compared to known segmentation methods. Moreover, the method is fast enough and permits user relevance feedback, meaning, it enables the physician to correct and refine segmentation result in an interactive and easy manner. Section 5 describes briefly the experimental system for testing and comparing the new segmentation method with methods known from the literature. The new segmentation method is tested over 100 cases of FISH images collected, prepared and evaluated from the Institute of Pathology, University of Bern, Switzerland. Results presented in Section 6 demonstrate the efficiency of the IMFA algorithm and its advantages compared to known methods. Some concluding remarks are presented in Section 7.

## Problem statement and related work

Segmentation, in general, is a challenging task in image processing. This process is typically used to locate particular objects in images, that is, to assign a label to every image pixel in such way that pixels with the same label share certain common characteristics. Particular attention is devoted to segmentation in medical images. Characteristic parts within a medical image, which are detected and extracted from the background, enable the identification of abnormalities and help physicians to perform the diagnosis. Depending on imaging technology and particular problem under analysis, different segmentation methods and techniques are derived and reported (Gonzales and Woods 2008; Suri et al. 2002). Regarding the HER2 scoring in FISH images, the first and most significant step is the cell nuclei segmentation, because only within well segmented nuclei the HER2 scoring has to be determined as recommended by ASCO/CAP (2013).

**Fig. 1:**
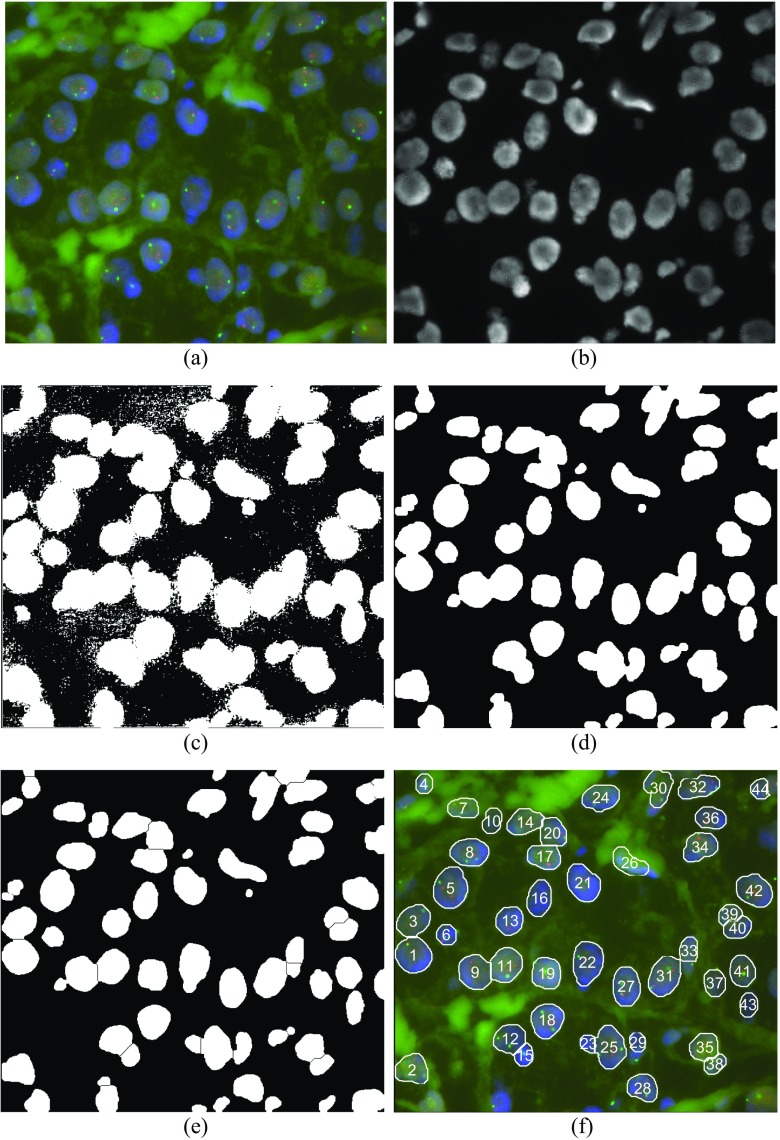
(**a**) FISH image 312,292.jpg. (**b**) Blue channel of an image. (**c**) Initial binarization after simple thresholding. (**d**) Refined binary image after morphology processing: hole filling (if necessary) and opening (small artifacts removal). (**e**) Separation of cell nuclei after applying distance- and watershed transform on Fig. 1(**d**). (**f**) Final result after postprocessing: rejection of small, non-oval and border regions, and nuclei enumeration

An example illustrating typical procedure for cell nuclei segmentation in FISH images is presented in Fig. 1. Initial FISH image (image stored as 312,292.jpg in our database) in RGB color space is depicted in Fig. 1(a). Within its blue channel, Fig. 1(b), nuclei are presented as well defined and well recognized oval regions brighter than surrounding. In this case the nuclei segmentation is relatively easy task: by applying simple thresholding the binary image (black and white) containing white regions (possible nuclei) and black background is obtained, as depicted in Fig. 1(c). By applying some morphology operations (hole filling and opening) on thresholded image, refined binary image as in Fig. 1(d) is produced: holes within nuclei regions (not existing in this case) are filled and small objects (artifacts) are removed.

An additional image processing tools, for instance, the distance- and watershed transform (Gonzales and Woods 2008), enable the separation of touched adjacent regions, as presented in Fig. 1(e). Some postprocessing steps for rejecting small, non-oval, and border nuclei are applied as well, and the final result is presented in Fig. 1(f). However, note that this case is very simple and needs not complex processing. Unfortunately, in everyday medical praxis, even after strictly and carefully applying the procedures recommended by ASCO/CAP (2013) obtained FISH images can be of degraded quality. For instance, due to finite thickness of histopathology samples within the microscopy field of view (FOV) obtained images may be blurred with no clear distinction of nuclei. Furthermore, the tissue staining is complex and sensitive procedure and images can be with unbalanced intensities, heterogeneous contrast, non-uniform color within the same tissue part, and with artifacts. As a consequence, cell nuclei segmentation may be difficult and incorrect. Additionally, manual dot scoring is fatiguing and time consuming process. For resolving such real-life problems and helping physicians in HER2 scoring, several automated or semiautomated methods have been proposed (Netten et al. 1997; de Solorzano et al. 1998; Kozubek et al. 1999; Lerner 2004; Raimondo et al. 2005), as will be briefly reviewed.

In their work Netten et al. (1997) considered automatic dots counting in lymphocytes from cultured blood. They used the ISODATA thresholding, introduced by Ridler and Calvard (1978), for separating nuclei from background. Dot detection within nuclei is performed by using the top-hat transform and a nonlinear Laplacian filter. De Solorzano et al. (1998) developed a method to segment nuclei in leukocytes in blood samples also using the ISODATA thresholding algorithm. After initial segmentation they used the watershed algorithm and the distance transform to isolate nuclei, and top-hat transform for dots detection. Similarly, Kozubek et al. (1999) described a system for analyzing FISH images in which the nuclei are segmented using bimodal histogram thresholding and a watershed-based algorithm. The use of Bayesian classifier for a FISH image classification system was considered by Lerner (2004). An efficient multistage algorithm for automated classification of FISH images from breast carcinomas biopsy specimens is described by Raimondo et al. (2005). Initial segmentation is based on non-linear processing with square root function, morphological opening, top-hat transform and Otsu algorithm (Otsu 1979) for global thresholding. Very interesting post-processing step based on geometric rule is applied to distinguish holes which appear within a nucleus from those between nuclei. The last step of nuclei segmentation in the algorithm proposed by Raimondo et al. (2005) involves the distance transform and watershed algorithm, to detect borders in overlapping nuclei clusters.

In all of these methods after initial binarization and segmentation an additional decision based on the cell’s morphology (considering shape, roundness, eccentricity, object area, etc.) may be applied for rejecting parts which belong to artifacts, as considered in papers by Malka and Lerner (2004); Lerner and Malka (2011).

**Fig. 2:**
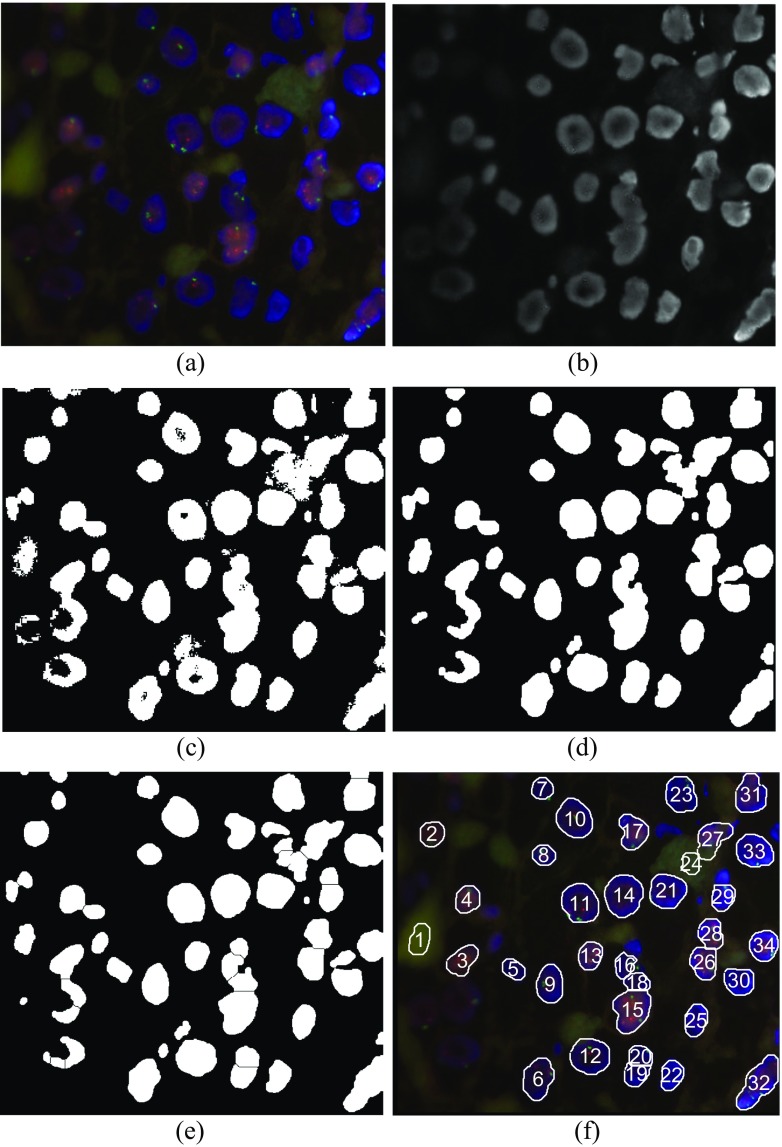
(**a**) FISH image 1,869,659.jpg. (**b**) Blue channel of an image. (**c**) Initial binarization after simple thresholding. (**d**) Refined binary image after morphology processing: hole filling (if necessary) and opening (small artifacts removal). (**e**) Separation of cell nuclei after applying distance- and watershed transform on Fig. 2(**d**). (**f**) Final result after postprocessing: rejection of small, non-oval and border regions, and nuclei enumeration

**Fig. 3:**
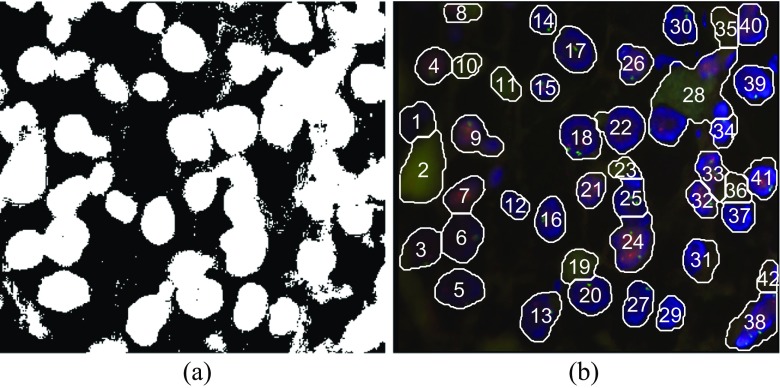
(**a**) Initial binarization of blue channel of FISH image 1,869,659.jpg after lowering the threshold level. (**b**) Final segmentation result after applying the procedure as in previous cases in Figs 1 and 2

In case as in Fig. 1 the cell nuclei segmentation is not difficult task. Unfortunately, as noted above, in medical praxis FISH images can be of degraded quality. One such example, FISH image 1,869,659.jpg from our database, is presented in Fig. 2(a). This image is characterized by low contrast on the left side and inhomogeneous brightness from left to right. From its blue channel, Fig. 2(b), it is obvious that nuclei zones are not well defined. Standard and advanced methods for nuclei segmentation, for instance, as proposed by Raimondo et al. (2005), and additional morphology postprocessing, are not efficient in this case – nuclei on the left side are not segmented, as depicted in Figs 2(c) to 2(f). Details within the left side region can be enhanced by lowering the threshold level, enabling in this way better nuclei recognizing on this side but the rest of image will be degraded: extremely large connected white areas appear on the right, as depicted in Fig. 3(a), and nuclei segmentation becomes difficult. Additional postprocessing does not improve the segmentation. The final result will be as depicted in Fig. 3(b). Now the region on the right, labeled by numeral 28, covers several nuclei.

Automated segmentation of FISH images seems very promising, but it suffers from at least two hard drawbacks as follows. First, it is difficult to automatically find an appropriate FOV within the biopsy slide. Furthermore, according to ASCO/CAP recommendations (2013) only the invasive component of a carcinoma should be assumed for HER2 scoring, without necrotic areas and cells with ambiguous border. Consequently, automated nuclei segmentation and machine cell distinguishing still are very difficult tasks as noted by Pajor et al. (2012). From these reasons, as an alternative way, it was suggested by Kozubek et al. (2001) to combine automated segmentation with human supervising and manual correction yielding more useful results.

## Fractals and multifractals

In his seminal paper Polish mathematician Benoit Mandelbrot (1967) coined the term *fractal*, for describing natural shapes (such as the coastline) which are characterized by a non-integer (i.e., fractional, or fractal) dimension. Introducing the concept of *fractal dimension* (FD) different complex phenomena, objects, and signals can be evaluated quantitatively, permitting thus their objective description, characterization and comparison. Fractal dimension numerically describes the property known as a *self-similarity*: the topological or geometrical properties of an object remain (almost) invariant at different scales. For a number of artificially generated structures by following some strict rules (as for instance, the Cantor set, the Von Koch curve, the Sierpinski gasket and/or blanket, etc.) the FD is exactly the same at all scales (in these cases the FD is called also a *similarity dimension*) – see Peitgen et al. (2004). Such objects are known as *monofractals*, since they are characterized by a single number – the FD. Conversely, a number of phenomena, shapes, or objects (particularly natural objects), are not strictly self-similar and are characterized by different FDs under different scales. Then, instead of single FD, a distribution of FDs over different scales can be observed, which is a concept of *multifractals*, as Mandelbrot noticed (Mandelbrot 1983, Mandelbrot 1989). Over last several decades fractal geometry and multifractal analysis have been accepted and applied as powerful methods for describing, evaluating, and comparing complex objects and phenomena. Among different applications these techniques have been found significant place in signal and image processing (Vehel 1996, 1998; Turner et al. 1998; Reljin et al. 2000).

Fractal dimension can be derived in different ways. Very popular algorithm for estimating the FD is the box-counting method described in Peitgen et al. (2004). In this method the observed structure is covered by a regular grid of boxes, *B*_*i*_, with a side length *ε* (assuming normalized space, i.e., *ε* ≤ 1). By counting the number of non-empty boxes *N*(*ε*), that means, counting boxes containing at least a part of observed structure, the box-counting dimension *D*_*b*_ can be estimated as

1 $$ {D}_b=-\underset{\varepsilon \to 0}{ \lim}\frac{ \ln \left(N\left(\varepsilon \right)\right)}{ \ln \varepsilon } $$

Serious limitation of box-counting dimension is that it relates only to the existence of the structure within boxes irrespective of the structure strength inside boxes. Regarding to image processing, it means that the box-counting method is applicable only to binary images characterized by two values of pixel intensities: 0 (black) and 1 (white). For gray scale images (with pixel intensities ranged from 0 to 1) the normalized measure *μ* characterizing in some way the signal intensity within the box is introduced (Vehel 1996, 1998). By considering the measure within the box, the coarse Holder exponent is derived as

2 $$ {\alpha}_i=\frac{ \ln \left(\mu \left({B}_i\right)\right)}{ \ln \varepsilon } $$

which can be assumed as the fractal dimension of the measure *μ* within the box *B*_*i*_. When *ε* tends to zero the coarse Holder exponent approaches to limiting value *α*, known as the *Holder exponent* at observed point

3 $$ \alpha =\underset{\varepsilon \to 0}{ \lim }{\alpha}_i. $$

The Holder exponent depends on the actual position within the image and describes local regularity (or singularity) at this point. In the whole image there usually are many points having the same value of Holder exponent. The regularity of the whole image structure can be described from the distribution of Holder exponent, i.e., by counting the number of boxes *N*_ε_(*α*_*i*_) containing particular va﻿lue of *α*_*i*_

4 $$ {f}_{\varepsilon}\left({\alpha}_i\right)=-\mathrm{In}\left({N}_{\varepsilon}\left({\alpha}_i\right)\right)/\mathrm{In}\left(\varepsilon \right) $$

**Fig. 4:**
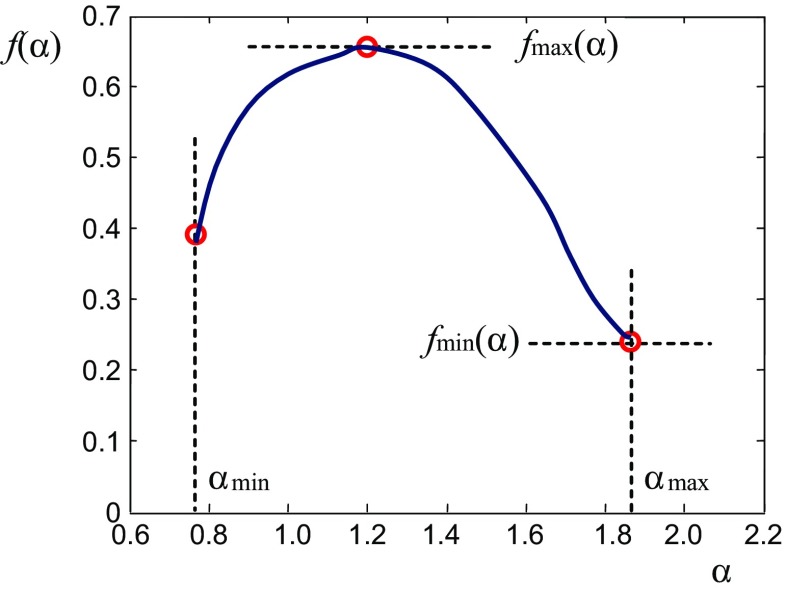
Typical shape of the multifractal spectrum

When *ε* → 0 the distribution *f*_*ε*_(*α*_*i*_) approaches to its limiting value *f*(*α*)

5 $$ f\left(\alpha \right)=\underset{\varepsilon \to 0}{ \lim }{f}_{\varepsilon}\left(\alpha \right) $$

which is known as the *multifractal spectrum* (or *singularity spectrum*). The MF spectrum describes the observed structure in a global sense. The function *f*(*α*) typically is parabola-shaped with limited values of *α* and *f*(*α*): α_min_, α_max_; *f*(α)_min_, *f*(α)_max_, as depicted in Fig. 4.

The MF quantities α and *f*(α), described by relations (2)–(5), are in connection with the generalized dimension *D*_*q*_ considered by Hentschel and Procaccia (1983) and Grassberger (1983), and can be used as signal (image) descriptors. For instance, in the paper of Theiler (1990) was stated that the value of α_min_ corresponds to the generalized dimension at *q* = +∞ (meaning that this value is related to the most dense points of observed object), while the value of α_max_, corresponds to its opposite. Similar conclusion was derived by Levy Vehel (1996, 1998) and he stated that:

1. The Holder exponent α describes local characteristics of observed signal:

- locally non-regular points (locally quite different from surrounding points) are characterized by high value of Holder exponent α,
- points within the smooth region (locally similar to surrounding) have small value of α.

2. The quantity *f*(α) relates to the distribution of exponent α and describes the signal globally:

- small value of *f*(α) relates to rare events (singularities within the signal) characterized by this value of α,
- points with high value of *f*(α) correspond to larger regions having similar local behavior described by α.

From these points it follows that by the pair (α,*f*(α)) both local and global regularity/singularity of the signal can be described simultaneously (Vehel 1996, 1998).

Several techniques for estimating the multifractal spectrum of observed structure are reported. From the practical point of view the determination of *f*(α) directly from experimental data, as introduced by Chhabra and Jensen (1989), is very useful and convenient. Based on their work, custom developed software is realized by Reljin et al. (2000), from which the *inverse multifractal analysis* (IMFA) is possible. The IMFA permits the bidirectional mapping from original signal space to multifractal space. Regarding to images, the IMFA means that from given intensity image **I** = {*I*(*m*,*n*)}, *m* = 1,2,…,*M*; *n* = 1,2,…,*N*, each pixel at position (*m*,*n*) can be characterized by appropriate values of α and *f*(α): α(*m*,*n*), *f*(*m*,*n*). In this way the two matrices: **A** = {α(*m*,*n*)} and **F** = {*f*(*m*,*n*)} can be created, with one-by-one correspondence with the image matrix **I** = {*I*(*m*,*n*)}. By choosing particular values of α or *f*(α) (say, α_p_, or *f*_p_) within matrices **A** or **F**, we can extract image pixels characterized just by these values α_p_ or *f*_p_, as shown in Reljin et al. (2000), i.e., we can extract image details having these particular local or global MF values. Throughout the further text the matrix **A** of Holder exponents will be denoted as an *alpha-image*.

## Multifractal-based cell nuclei segmentation

The MF concept was applied successfully in image analysis in general (Vehel 1996, 1998; Turner et al. 1998; Reljin et al. 2000), as well as in medical image segmentation and classification (Stojic et al. 2006; Huang and Lee 2009; Vasiljevic et al. 2012; Baravalle et al. 2015) and in texture description (Xia et al. 2006). Our research is addressed to possible application of MF analysis to nuclei segmentation in FISH images, particularly for degraded quality images, such as the case in Fig. 2. By analyzing the blue channel of FISH images from our database we are faced with interesting result. Namely, although subjectively this is not so obvious (due to the nonlinearity of the human visual system (Gonzales and Woods 2008)), we found that nuclei zones are characterized by relatively low local contrast not exceeding 2:1 (assuming maximum vs. minimum pixel intensities within these regions), while the local contrast within the background always was higher – of order 3:1 or more. Since nuclei regions are small-sized regarding to the whole image, these regions are characterized by small local changes (i.e., these regions are with dense pixels with similar intensities) and thus, from the multifractal point of view (Theiler 1990; Vehel (1996, 1998)), is expected that these regions have small values of Holder exponent α.

**Fig. 5:**
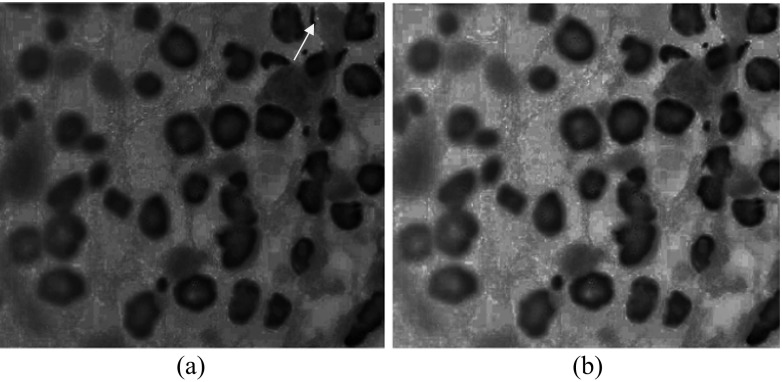
(**a**) Image of Holder exponents (alpha-image) of the blue channel of image 1,869,659.jpg, rescaled to the full range (0–1). Two isolated singularities on the upper right (having maximal value of 1, i.e., visible as white dots) produce the rest of image as dark gray. (**b**) The new rescaled alpha-image after changing the value of these two isolated singularities to 0.5

For approving this assumption let us consider again the image 1,869,659.jpg as in Fig. 2. From its blue channel we calculated Holder exponents by custom developed software (Reljin et al. 2000) and found that the whole image is characterized by Holder exponents ranged from α_min_ = 0.89404 to α_max_ = 1.5382. Corresponding alpha-image, with rescaled values of Holder exponents from α_min_-α_max_ to the range 0–1 prior to visualization, is depicted in Fig. 5(a). In this image all nuclei regions (irrespective of their actual intensity level in the blue channel) are presented with quite similar dark-gray levels. Note that in this example several pixels are highly singular, having highest value of α, α = α_max_ (i.e., having maximal intensity of 1 in rescaled alpha-image), and are presented as white dots (labeled by an arrow in upper right). These isolated pixels produce the rest of image becomes dark gray. By changing the value of these pixels to half tone (value of 0.5) and creating the new alpha-image in the full range 0–1, the result as in Fig. 5(b) is obtained, with better visualization of nuclei regions.

**Fig. 6:**
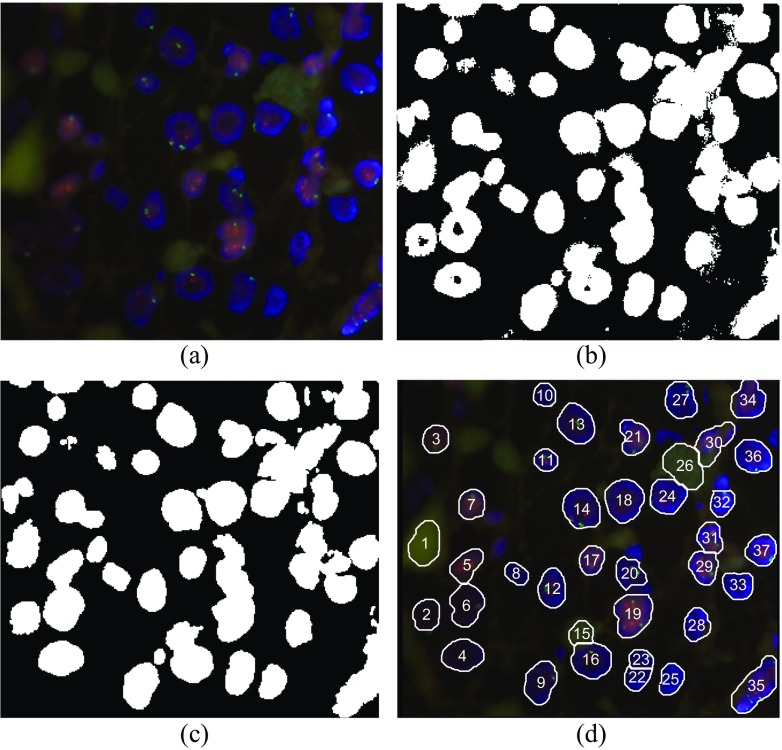
Illustration of nuclei segmentation by using multifractal approach. (**a**) Input image 1,869,659.jpg. (**b**) Initial binarization of original image after hard thresholding with α_T_ = 1.1·α_min_ applied to alpha-image as in Fig. 5(**a**). (**c**) Refined image after some processing steps (hole filling and opening). (**d**) Final result after nuclei separation, rejecting small and boundary regions, and nuclei labeling

For given example as in Fig. 5(a) the non-rescaled values of Holder exponent within nuclei regions are ranged from α_min_ = 0.89404 to α = 0.98865, which is approximately 1.1·α_min_, while for the rest of image Holder exponents take greater values. From that point we found that successful initial binarization (initial nuclei selection) can be obtained by simple threshold: parts in alpha-image having α > α_T_ = 1.1·α_min_ should be black (background) otherwise are white (representing possible nuclei). Holes which can arise within several nuclei and small artifacts in the background can be easily removed by applying some morphology operators (hole filling and opening). The whole procedure is illustrated in Figs 6(a) to 6(d). It is obvious that despite the low contrast on the left side of original image 1,869,659.jpg and inhomogeneous brightness from left to right side, the MF-based segmentation is quite efficient, enabling successful nuclei extraction within the whole image.

After intensive simulations we found similar result for all FISH images from our database: nuclei regions in alpha-images are characterized by low values of Holder exponent. By hard thresholding with the threshold α_T_ close to α_min_ initial nuclei segmentation was quite successful in all cases. For our dataset by using α_T_ within the range 1.05·α_min_ ≤ α_T_ ≤ 1.14·α_min_ (depending on particular image) an efficient initial segmentation was obtained. Note that from once determined alpha-image we can correct easily the sensitivity of initial segmentation by changing only the threshold value α_T_ in an interactive manner. By using higher threshold more details will be selected as white (possible nuclei) and vice versa. The user observes the initial segmentation and can refine segmentation. If the user is satisfied with initial (or refined) segmentation the image can be further processed – red/green dots detection and calculation of HER2 positivity. From our research we suggest the new method for cell nuclei segmentation based on the IMFA as summarized in Table 1.

**Table 1:** Algorithm for nuclei segmentation based on the multifractal approach

Step	Procedure
1.	Extract the blue (B) image from initial RGB FISH image
2.	Find multifractal spectrum of the B image
3.	Create an alpha-image
4.	Define the threshold in alpha-image. Default value is α_T_ = 1.1·α_min_
5.	Apply the inverse MF analysis with hard thresholding:
	Image pixels with α > α_T_ should be black (background), otherwise are white (possible nuclei)
6.	Inspection and decision by the user:
	If initial segmentation is satisfactory, continue to step 7
	Otherwise correct the threshold value α_T_ and return to step 4
7.	Postprocessing: rejecting small, non-oval, and border regions
8.	Segmentation of adjacent and overlapped nuclei
9.	Final inspection by user. Additional rejecting regions which are not nuclei or not belonging to the invasive component of a carcinoma, according to ASCO/CAP recommendations

## Experimental system

**Fig. 7:**
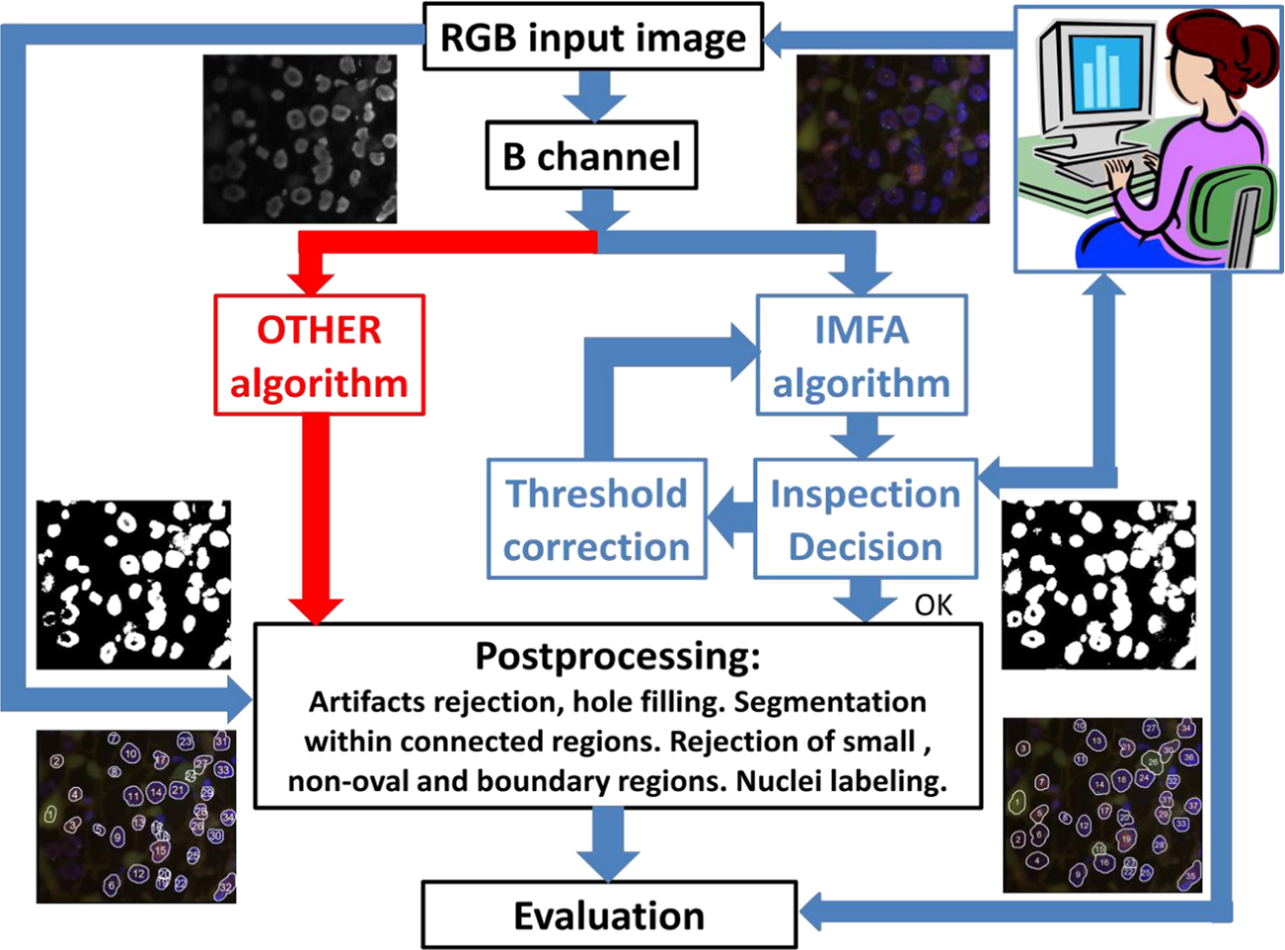
Block scheme of the experimental system for nuclei segmentation based on the IMFA algorithm. Segmentation results are compared with those obtained by other known methods denoted here as the OTHER algorithm

In order to test and evaluate proposed algorithm the experimental system as in Fig. 7, is realized. The whole procedure is semiautomatic and is performed in two basic steps: (*i*) initial segmentation is automatic while (*ii*) the user feedback is applied for refining the segmentation. The FISH images in RGB format are manually selected from database and uploaded to the system. Segmentation is derived from the blue (B) channel of an image by applying the new IMFA method briefly described in Table 1. Initial segmentation (image binarization) is realized according to step 5 in Table 1 and the result is inspected by the user. If initial segmentation is not appropriate, the user can correct the threshold α_T_: by using higher value of α_T_ more details will be selected, and vice versa with lower value of α_T_. When a user is satisfied with initial segmentation, the binary image is postprocessed, according to step 7 in Table 1: artifacts (small details) are rejected and holes are filled. Segmentation within connected regions (step 8 in Table 1) is performed by using distance transform and watershed transform. Small, boundary, and non-oval shaped regions are rejected automatically, as well. Additionally, user can reject parts not satisfying ASCO/CAP recommendations (step 9). Then, remaining parts (nuclei) are labeled and their contour lines are superimposed over initial RGB image. Within segmented nuclei the HER2 status can be derived by counting red and green dots and finding their ratio.

For comparison purposes the same images are processed by following some other, already reported procedures and methods, for instance the procedure proposed by Raimondo et al. (2005), and similar. In future text we will denotes our method as “IMFA algorithm” and other known procedures as “OTHER algorithm”. Obtained segmentation results, after applying both methods, are presented to skilled pathologist for evaluation.

## Testing results and discussion

The new segmentation method based on the IMFA is tested and evaluated over FISH images prepared and collected from the Institute of Pathology, University of Bern, Switzerland. The assessment of the HER2 gene status was performed using a FDA approved commercially available kit including a dual-color FISH probe (PathVysion®; Abbott /Vysis, Downers Grove IL, USA). The HER2 gene DNA is stained in orange (Spectrum Orange) and the centromeric probe 17 in green (Spectrum Green). Five μm sections were cut from a paraffin embedded tumor tissue block. Afterwards, the slides were deparaffinized, air dried, and rehydrated followed by a pretreatment step (with pretreatment solution Vysis®) for 30 min at 80 °C. The slides were incubated with protease (Vysis®) for 25 min at 37 °C, and afterwards washed and dehydrated through graded alcohols and air dried. Then, the slides were denatured for 2 min at 85 °C. The hybridization with the FISH probes was performed in a humid chamber at 37 °C for 14 h. After hybridization the slides were washed, air dried, counterstained with DAPI and covered with a cover slip.

The slides were analyzed with a Zeiss Axioskop 2 (Carl Zeiss, Jena, Germany) equipped with a filter set for DAPI, Spectrum Orange and Spectrum Green (Vysis®). Representative images were digitized and taken with a magnification of 630× (10× (electronic) x 63× (optical - objective NEOFLUAR 63X)) using an AxioCam MRm camera (Zeiss, Jena, Germany) and the Isis FISH imaging system software (V5.1.5.). Digitized images were stored in RGB format with resolution of 1016 × 896 pixels.

From the whole image dataset physicians selected 100 samples satisfying the ASCO/CAP recommendations (2013). These images were processed and analyzed by using the system as in Fig. 7. Segmentation results after the IMFA and OTHER algorithm are evaluated by skilled pathologist assuming their manual segmentation as a true (T) value. After evaluation, the segmentation accuracy for each image from our database is calculated as a ratio of the number of segmented nuclei by using the IMFA (I) and OTHER (O) algorithm vs. the number of manually segmented nuclei (T):

6 $$ \mathrm{Accuracy}=\left\{\kern0.75em \begin{array}{c}\frac{I}{T},for\ \mathrm{IMFA}\ \mathrm{algorithm}\\ {}\frac{O}{T},for\ \mathrm{OTHER}\ \mathrm{algorithm}\end{array}\right. $$

**Fig. 8:**
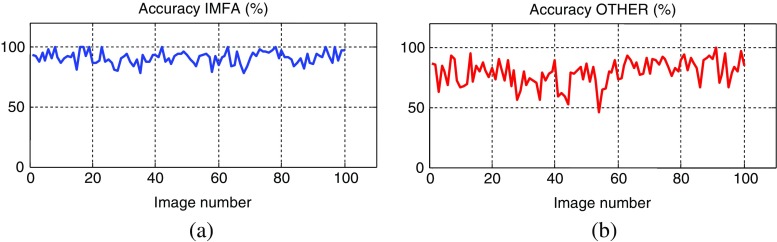
Segmentation accuracies (in percents) for the two algorithms: (**a**) IMFA and (**b**) OTHER. The means and standard deviations respectively are: 90.86% and 0.0541 for the IMFA algorithm, and 79.10% and 0.1062 for the OTHER algorithm

**Fig. 9:**
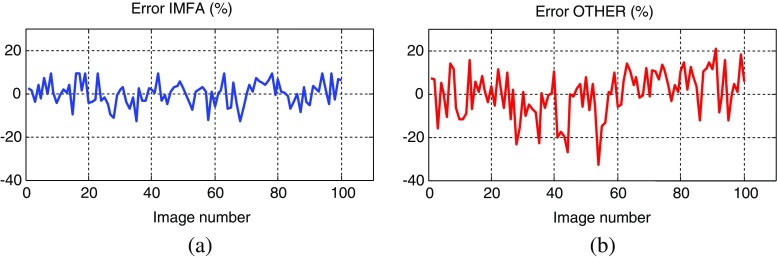
Relative errors (in percents) for the two algorithms: (**a**) IMFA and (**b**) OTHER, calculated as deviations of accuracies from their means

Segmentation accuracies (in percents) for particular images (samples) denoted by numerals 1–100 are plotted in Fig. 8, while in Fig. 9 the plots of relative errors, calculated as the deviation of accuracies from their means, are depicted.

Obtained results indicate to efficiency of proposed algorithm based on the inverse multifractal analysis, denoted here as the IMFA algorithm. For the whole dataset of 100 FISH images from our database the mean accuracy of the segmentation was 90.86% with the standard deviation of 0.0541. Relative error was within limits +9% to −13%. These results are quite better than those obtained by simulating already reported procedures (mainly inspired by Raimondo et al. (2005)) denoted here as the OTHER algorithm. By applying the OTHER algorithm on the same dataset the mean and standard deviation are 79.10% and 0.1062, while relative error was within the limits +21 to −33%. From these results the new IMFA algorithm seems to be a promising tool for nuclei segmentation in FISH images.

## Conclusion

In this paper the new method for nuclei segmentation in FISH images is proposed. The method is based on the inverse multifractal analysis applied within the blue channel of FISH images stored in RGB format. The method is semi-automatic with the user’s feedback. Initial image binarization is automatic: from the blue channel of FISH image regions characterized by low values of Holder exponent (lover than initially defined threshold α_T_) are remapped to white (possible nuclei regions), otherwise are black (background), and obtained binary image is displayed on the screen. The user (skilled pathologist) observes the result and makes the correction of initial segmentation, if necessary, by changing the threshold level. Except the correction of initial segmentation, the user’s feedback can be applied for additional fine tuning as well. Since automatic (or corrected) segmentation may extracts regions not belonging to nuclei or not belonging to the invasive component of a carcinoma, the user can remove these regions manually, enabling more accurate segmentation.

The new IMFA algorithm was tested over FISH images from clinically prepared and collected cases in the Institute of Pathology, University of Bern, Switzerland. Obtained results are very promising: for the dataset of 100 FISH images the mean accuracy of the segmentation was 90.86% with the standard deviation of 0.0541. These results are better than those obtained on the same dataset by applying already reported methods denoted here as OTHER algorithm: the mean accuracy and standard deviation are 79.10% and 0.1062 by using the same dataset.
